# Young-onset dementia following chronic abuse of amphetamine-type stimulants

**DOI:** 10.12669/pjms.40.4.8737

**Published:** 2024

**Authors:** Sultan H. Alamri

**Affiliations:** i Sultan H. Alamri, MBBS Department of Family Medicine, Faculty of Medicine, King Abdulaziz University, Jeddah, Saudi Arabia; ii Neuroscience and Geroscience Research Unit, King Fahad Medical Research Center, King Abdulaziz University, Jeddah, Saudi Arabia; iii Geriatric Medicine Service, Doctor Soliman Fakeeh Hospital, Jeddah, Saudi Arabia; iv Saudi Geriatrics Society, The National Center for the Development of the Non-Profit Sector, Riyadh, Saudi Arabia

**Keywords:** Amphetamine, Methamphetamine, Alzheimer’s disease, Dementia, Cognition

## Abstract

Young-onset dementia (YOD) is influenced by various risk factors, including substance abuse. In this report, we present the case of a 54-year-old man who developed YOD following prolonged abuse of amphetamine-type stimulants. The patient exhibited insidious cognitive decline over a three-year period before seeking medical attention. Neuroimaging revealed atrophy of the temporal lobe, suggesting a connection between amphetamine-induced neurotoxicity and the cognitive abnormalities observed in the patient condition. Our case highlights the importance of considering amphetamine-type stimulants as potential risk factors for YOD and emphasizes the need to recognize cognitive impairment resulting from substance abuse. Additionally, we look into relevant literature to provide further context and insights.

## INTRODUCTION

Young-onset dementia (YOD) is a distinct form of dementia occurring before the age of 65 years, carrying significant implications for patients and caregivers. It demands increased attention to its diagnosis, prognosis, and treatment. YOD accounts for approximately 5% of all dementia cases, with an estimated incidence of 119 cases per 100,000 individuals.^1^ Common YOD causes include Alzheimer’s disease, vascular dementia, and frontotemporal lobar degeneration, whereas Alzheimer’s disease represents approximately 30%-40% of cases.^2^ Research into YOD heritability underscores the role of polygenic risk scores, where a combination of genetic variants contributes to susceptibility. Although genetic factors lay the foundation, interactions between genes and the environment are at the forefront.^3^

Rigorous investigation into environmental influences has unveiled a complex network of interconnected variables associated with YOD. Lifestyle choices, such as physical inactivity, poor diet, smoking, alcohol abuse, and drug intoxication, have emerged as modifiable risk factors contributing to YOD development.^4^,^5^ The surge in the prevalence of amphetamine-type stimulant (ATS) abuse, involving amphetamines (amphetamine and methamphetamine) and substances within the “ecstasy” group, has elevated these substances to a global public health concern impacting millions worldwide. The prevailing perspective posits that ATS abuse is associated with significant cognitive impairments.^6^ However, to date, only one case of amphetamine-related dementia has been reported.^7^ Herein, we present the case of a middle-aged man with YOD following a prolonged history of ATS abuse and provide a comprehensive literature review.

## CASE REPORT

The patient, a 54-year-old retired male security officer, was referred to a geriatrics clinic for evaluation due to an insidious onset of cognitive decline. As reported by his family, patient symptoms began three years before the visit and included forgetfulness of recent information, misplacement of items, difficulties in learning new tasks, and impaired attention. His medical history was notable for dyslipidemia. The patient was not on any medications and had no history of head trauma or injury. No family history of neuropsychiatric disorders existed. He was a current smoker, consuming one pack of cigarettes daily. He initiated oral and nasal ATS abuse in his 20s, with no specified quantities mentioned. This pattern continued until his late 40s when he successfully quit after experiencing multiple relapses.

During the examination, patient blood pressure measured 125/86 mmHg, with a regular heart rate of 90 beats/minute. A mental status examination revealed sporadic eye contact, appropriate affect, slightly disorganized thoughts, limited insight into his condition, and poor judgment. No abnormal movements were observed. On cognitive testing, he scored 0 on the Mini-Cog test including 0 out of 3 on the three-word recall task. Additionally, the patient demonstrated reduced verbal fluency and poor calculation ability. Neurological examination revealed normal cranial nerve function, muscle strength, and tone, with normal deep tendon reflexes. Sensation was symmetrical and intact. His cerebellar function was normal, and no gait abnormalities were observed. Other systemic examinations yielded unremarkable results.

Laboratory tests, including complete blood count, liver function, thyroid function, urea, creatinine, electrolytes, vitamin B12, C-reactive protein level, erythrocyte sedimentation rate, and urine analysis all fell within the normal ranges. A lipid panel revealed a total cholesterol level of 5.6 mmol/L, triglyceride level of 5.3 mmol/L, and low-density lipoprotein level of 3.44 mmol/L. Serological tests for hepatitis B and C and human immunodeficiency virus were negative.

**Fig.1:**
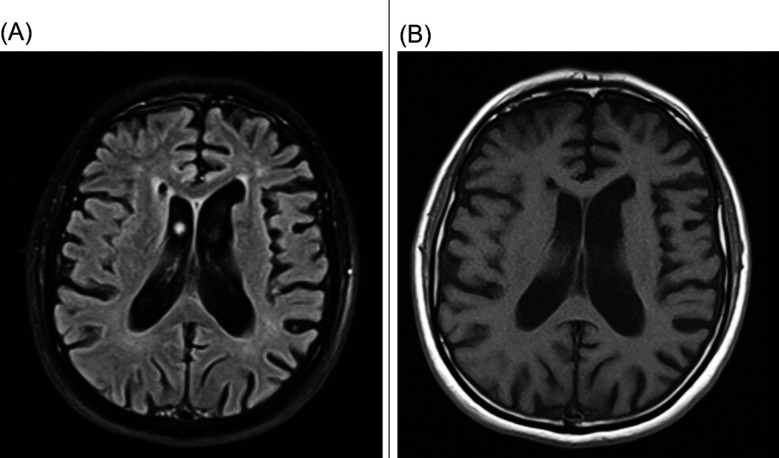
Brain magnetic resonance imaging revealing bilateral temporal lobe atrophy. A) Fluid attenuated inversion recovery. B) T1-weighted axial sections.

Brain magnetic resonance imaging revealed generalized diffuse prominence of the cortical sulci, cerebrospinal fluid spaces, and ventricular system, as well as small confluent abnormal T2 and fluid-attenuated inversion recovery hyperintensities affecting the corona radiata of both cerebral hemispheres. The most significant atrophic changes were observed in the temporal lobes, with notable ballooning of the temporal horns of the lateral ventricles (Fig.1). YOD was diagnosed based on clinical manifestations and diagnostic tests.

## DISCUSSION

Several studies have examined the connection between drug abuse and dementia, with a specific emphasis on substances such as ATS. Amphetamines, particularly methamphetamine, when used over extended periods or at high doses, can lead to brain toxicity and affect peripheral organs. This phenomenon is more pronounced in regions rich in nerve connections within the sympathetic nervous system.^8^

Episodic memory impairment has been linked to methamphetamine abuse, potentially contributing to recurrent drug use. In a nationwide retrospective cohort study in Taiwan involving 68,000 participants followed for 15 years, amphetamine-related disorders were strongly associated with dementia. The amphetamine-related disorder cohort had nearly a fivefold increased risk of developing dementia, including Alzheimer’s disease and vascular dementia. This association persisted after adjusting for age, sex, education, and comorbidities.^9^

Another study examined the effects of amphetamine and heroin abuse on cognitive function. Various neuropsychological tests, including visual discrimination learning, conventional tests, and computerized assessments for recognition and spatial working memory, were conducted. Both the amphetamine and heroin groups experienced significant impairment in a pattern recognition memory, which is sensitive to temporal lobe dysfunction. This suggests that chronic amphetamine and heroin use may result in distinct patterns of cognitive impairment.^10^

Ersche et al. assessed the effects of chronic amphetamine and opiate use on the cognitive function in current and previous users in the United Kingdom. This study focused on executive and memory function as indicators of neurocognitive measures. Participants demonstrated notable impairment in cognitive domains such as the Tower of London planning task, paired associate learning, and pattern recognition memory. However, amphetamines users displayed more severe cognitive impairment than current opiate users. The study concluded that chronic users of amphetamines and opiates experienced lasting impairments in memory and executive function, even after years of discontinuation.^11^

Wang et al. conducted a cross-sectional survey in Guangdong Province, China, involving 528 methamphetamine users undergoing detoxification and rehabilitation. Cognitive impairment was assessed using the Montreal Cognitive Assessment Scale, revealing that approximately 69.89% of participants experienced cognitive impairments. Additionally, advanced age (≥30 years), prolonged duration of drug use (>2 years), and daily consumption of methamphetamines were associated with more severe cognitive impairment. The study firmly concluded that chronic methamphetamine use leads to cognitive impairment.^12^

The sole published case report on amphetamine-related dementia from Australia involved a 48-year-old Australian man who had been using amphetamines for approximately 30 years.^7^ This patient struggled with maintaining task continuity and frequently experienced lapses in remembering appointments, names, and familiar faces. More pronounced impairments were observed in the memory and verbal fluency domains than in the attention and orientation domains.^7^

## CONCLUSION

In conclusion, amphetamine abuse is associated with the deterioration of cognitive function across multiple domains. This less-recognized potential cause of cognitive dysfunction may become more prevalent in the future, highlighting the importance of considering ATS as risk factors for dementia. Healthcare professionals must identify and provide appropriate care to individuals experiencing cognitive dysfunction resulting from substance abuse.
